# Pharmacological and interventional treatment of benign prostatic obstruction: An evidence‐based comparative review

**DOI:** 10.1002/bco2.74

**Published:** 2021-02-03

**Authors:** Sachin Malde, Wayne Lam, Zainal Adwin, Hashim Hashim

**Affiliations:** ^1^ Department of Urology Guy’s and St Thomas’ NHS Foundation Trust London UK; ^2^ Division of Urology Department of Surgery LKS Faculty of Medicine Queen Mary Hospital The University of Hong Kong Hong Kong Hong Kong SAR; ^3^ Department of Surgery Faculty of Medicine Universiti Teknologi MARA Selangor Malaysia; ^4^ Bristol Urological Institute Southmead Hospital North Bristol NHS Trust Bristol UK

**Keywords:** Aquablation, benign prostatic obstruction, benign prostatic enlargement, embolization, laser, lower urinary tract symptoms, pharmacotherapy, Rezum, TURP, Urolift

## Abstract

**Introduction:**

The recommended treatment for bothersome lower urinary tract symptoms (LUTS) secondary to benign prostatic obstruction (BPO) after the failure of behavioral therapy and fluid modification includes pharmacological, minimally invasive interventional, and surgical approaches. Each option has different risk and benefit profiles, and the urologist must be aware of the unique characteristics of each option in order to be able to accurately counsel the patients based on their individual values and preferences. We provide a comparative review of the commonest pharmacological and most widely performed interventional/surgical treatments for BPO, discussing the evidence for the treatment characteristics that are most useful for the practicing urologist.

**Methods:**

A search of the PubMed database was performed for articles reporting on the following treatments for LUTS due to BPO: α‐blockers, 5α‐reductase inhibitors, phosphpdiesterase‐5 inhibitors, prostatic urethral lift (Urolift), convective radiofrequency water vapor thermal therapy (Rezum), Temporary implantable Nitinol Device (iTIND), prostate artery embolization (PAE), transurethral resection of the prostate (TURP), photoselective vaporization of the prostate (PVP), Aquablation, and anatomical endoscopic enucleation of the prostate (AEEP). We performed a narrative review focussing on the following outcomes: efficacy, safety, durability, duration of catheterization, length of stay, re‐treatment rate, efficacy in special situations (enlarged median lobe, prostate size, urinary retention, and anticoagulant use), and sexual adverse events.

**Results:**

AEEP offers the greatest long‐term improvement in maximum flow rate, IPSS, and prostate volume reduction, with lowest re‐treatment rate, followed by PVP, TURP, and Aquablation. Urolift, Rezum, and PAE have similar efficacy for prostate volume up to 80cc, and all are more effective than the pharmacological treatment. Urolift offers the lowest rate of sexual dysfunction, followed by Rezum, and both can be performed as a day case under local anesthesia.

**Conclusion:**

Several treatment options exist to treat voiding LUTS due to BPO. Newer minimally invasive treatments reduce the hospital stay and postoperative complications, whereas AEEP provides the greatest long‐term symptom improvement at the expense of higher morbidity and sexual dysfunction. Men should be counseled regarding all suitable treatment options as some may favor reduced efficacy in association with reduced side effects.

## 1 INTRODUCTION

The management of bothersome lower urinary tract symptoms (LUTS) due to benign prostatic obstruction (BPO) consists of behavioral and dietary modifications, pharmacological therapy, and interventional approaches.^1^ Pharmacological treatment has traditionally been the mainstay of initial management for men with bothersome voiding LUTS once conservative measures have failed. Interventional or surgical treatment is typically recommended for men who have not responded to, or are unable to take (due to contraindications, adverse events, or personal preference), pharmacological treatments. However, there is increasing evidence that long‐term pharmacological treatment for BPO is associated with significant cognitive and psychiatric side effects (such as dementia an depression), and so men should be thoroughly counseled about these long‐term risks prior to commencing treatment.^2^ In recent years, advances in technology have led to the development of several novel and minimally invasive interventional treatments for LUTS secondary to BPO, with the aim of reducing morbidity, complications, and length of hospitalization compared to the current standard of care—transurethral resection of the prostate (TURP). Each modality offers unique risk/benefit profiles and has led to increased treatment choice for patients. In view of potentially serious long‐term consequences associated with the pharmacological treatment, men may prefer interventional treatment as an alternative to long‐term pharmacological therapy. As a result, the urologist must be aware of the evidence for the efficacy, safety, and unique characteristics of each option so that patients can be optimally counseled based on their individual values and preferences.

Treatments for BPO must demonstrate evidence of efficacy in different clinical scenarios, safety, reproducibility, and durability.^3^ We provide a comparative review of the commonest pharmacological and most widely performed interventional/surgical treatments for BPO, discussing the evidence for the treatment characteristics that are most useful for the practicing urologist (Tables 1 and 2).

**TABLE 1:** Summary of efficacy of BPO treatments

Treatments	Mean improvement in IPSS score (%)	Mean improvement in Qmax (%)	Mean improvement in post‐void residual (%)	Mean reduction in prostate volume (%)	Evidence of long‐term (>5 year) efficacy?	Histology available
α‐blockers	45	15‐30	50	0	No—high discontinuation rate	N/A
5α‐reductase inhibitors	30	30‐40	34	18‐29	No—high discontinuation rate	N/A
PDE5i	NR	NR	NR	0	No	N/A
TIND	50‐60	57‐101	NR	0	No	No
PAE	51‐69	50‐89	77	26	No	No
Rezum	45‐60	44‐72	20‐38	NR	No	No
Urolift	43	50	20	0	Yes	No
TURP	70	162	77	54	Yes	Yes
Aquablation	74	115	57	65	No	No
PVP	70	170	84	55	Yes	No
AEEP	80	277	92	70‐80	Yes	Yes

Abbreviations: AEEP, anatomical endoscopic enucleation of the prostate; and NR, not reported; PAE, prostate artery embolization; PDE5i, phosphodiesterase‐5 inhibitors; PVP, photoselective vaporization of prostate; TIND, temporary implantable nitinol device; TURP, transurethral resection of prostate.

**TABLE 2:** Summary of key characteristics of BPO interventional treatments

Property	PAE	TIND	Urolift	Rezum	TURP	Aquablation	PVP	AEEP
Transfusion (%)	0‐1	0	0	0	2	1.4%‐2.5%	0.2	0
Bladder neck contracture/urethral stricture (%)	0	0	0	0	5	0	4	5
Stress urinary incontinence (%)	0‐1	0	0	0	0.5‐1	0	0.5‐1	1‐5
Mean duration of catheterization (days)	0	0	0‐1	5‐7	2.5	2	1	1
Mean length of hospitalization (days)	0	0	0	0	3.6	1.5	0‐1	0‐1
Ejaculatory dysfunction (%)	12	0	0	0‐3	66‐75	0 to 32	22‐67	75
Erectile dysfunction (%)	0‐1	0	0	0‐3	6.5	0	6.5	0
Re‐treatment rate (%)	20 (at 2 years)	10 (at 2 years)	6% per year	4.4% (at 4 years)	13 (at 8 years)	3% (at 1 year)	10 (at 2‐3 years)	0% (at 7 years)
Can be performed under local anesthesia?	Yes	Yes	Yes	Yes	No	No	No	No
Efficacy in large (>80g) prostate	Moderate	No	Moderate (up to 100g)	Under investigation	Moderate	Yes	Yes	Yes
Efficacy for enlarged median lobe	Yes	No	Yes	Yes	Yes	Yes	Yes	Yes
Efficacy in acute urinary retention	Yes	No	Yes	Yes (but require prolonged catheterization)	Yes	Yes	Yes	Yes

Abbreviations: AEEP, anatomical endoscopic enucleation of the prostate; and NR, not reported; PAE, prostate artery embolization; PVP, photoselective vaporization of prostate; TIND, temporary implantable nitinol device; TURP, transurethral resection of prostate.

## 2 PHARMACOTHERAPY (NOT INCLUDING PHYTOTHERAPY)

### 2.1 α1‐Antagonists

BPO has two important components associated with it. The dynamic component is associated with an increased smooth muscle tone in the prostate and bladder neck. This increase is mediated by α1‐adrenoceptors.^4^ α1‐antagonists blocks these adrenoceptors causing relaxation of prostate smooth muscle thus modifying the dynamic component in BPO. Uroselectivity refers to the higher affinity of drugs toward the α1A‐adrenoceptors. These receptors are primarily responsible for contraction of the prostate smooth muscle. Newer α1‐antagonists have higher affinity for α1A‐adrenoceptors which allows high‐affinity binding to these receptors located in the prostate and bladder neck (Table 3).^5^

**TABLE 3:** Receptor selectivity of common pharmacological agents used to treat LUTS due to BPO

Drug	Type
Alfuzosin	Long acting α‐1a blocker (selective)
Tamsulosin	Long acting α‐1a blocker (selective)
Silodosin	Long acting α‐1a blocker (selective)
Doxazosin	Long acting α‐1 blocker
Terazosin	Long acting α‐1 blocker
Finasteride	5‐α reductase receptor type 2 &3 blocker
Dutasteride	5‐α reductase receptor type 1,2,&3 blocker
Tadalafil	Phosphodiesterase Type 5 inhibitor

#### 2.1.1 Efficacy

The efficacy among different types of α1‐antagonists has been published widely. These therapies improve the voiding aspects of the International Prostate Symptoms Score by up to 45% with an improvement in the quality of life (QoL) scale by 1‐1.5 points.^6^, ^7^ These medications are also able to improve the maximum urinary flow rate (Qmax) by 15%‐30% or 2‐3 mL/s.^8^ Jardin and co‐workers showed that α‐antagonists are able to improve post‐void residual volume by 50% compared to placebo.^9^ α1‐Antagonists have rapid onset and patients experience therapeutic improvement within 1 week.^10^ Despite having a rapid onset, its long‐term efficacy is still inconclusive and discontinuation rates are high.^11^ The Alfuzosin Long‐Term Efficacy and Safety Study (ALTESS) showed maintained significant symptom score and flow rate improvements for up to 2 years, but the Medical Therapy of Prostatic Symptoms (MTOPS) randomized trial showed deterioration in efficacy of doxazosin after 2 years in terms of risk of urinary retention or need for surgery.^12^, ^13^ Analysis of population‐based administrative databases have reported that only 23%‐35% are still taking α‐antagonists at 1 year after commencing treatment, and this reduces to 30% at 2 years, 24% at 3 years, and 19% at 4 years.^11^, ^14^

The urodynamic effects of α1‐antagonists have also been studied in Japan with both studies showing reductions in the detrusor pressure at maximum flow (Pdet Qmax) of approximately 20‐30 cm H_2_O, and in the Bladder Outlet Obstruction Index (BOOI) of approximately 30‐40, which are nearly commensurate with the effect of surgical intervention.^15^, ^16^

#### 2.1.2 Special situations

##### Acute Urinary Retention and Trial Without Catheter

The utility of the α1‐antagonists, alfuzosin, and tamsulosin, in acute urinary retention (AUR) and to improve the outcome of trial without catheter (TWOC) has been shown in a Cochrane review of five randomized trials. They concluded that these α1‐antagonists increase success rates of TWOC.^17^

#### 2.1.3 Adverse effects

Due to the multi‐locality of α1‐adrenoceptors, antagonizing these receptors are associated with a wide range of systemic adverse effects. The most widely noted side effect of α1‐antagonists is postural hypotension.^13^ Other effects include dizziness (5.3%), headache (less than 2%), asthenia (less than 2%), rhinitis (less than 2%), and sexual dysfunction.^18^ Sexual dysfunction has been related to ejaculatory disorders (retrograde or anejaculation), but is also postulated as a proxy of efficacy.^19^ Sexual dysfunction more common in uroselective α1‐antagonists due to their concentrated action in the lower urinary tract.^20^ Recent studies have reported an association between long‐term use of α1‐antagonists and risk of dementia, with the greatest risk reported for tamsulosin.^21^ Men should be counseled about the potential long‐term consequences prior to commencing treatment.

### 2.2 5α‐reductase inhibitors (5‐ARI)

The enzyme 5α‐reductase converts testosterone to DHT which stimulates an increase in prostate volume. Inhibition of the 5α‐reductase enzyme reduces this androgenic stimulation ultimately causing reduction in prostatic volume.^14^ There are two 5α‐reductase isoenzymes and the most predominant in prostatic stroma is the type 2 isoenzyme. The two most commonly used 5‐ARI inhibits the type 2 isoenzyme with dutasteride having some type 1 isoenzyme inhibition as well.

#### 2.2.1 Efficacy

Longer‐term study of finasteride at 36 months of therapy reported a 40% increase in Qmax.^22^ Men with larger prostate glands benefit more with finasteride monotherapy.^23^ The Proscar Long‐Term Efficacy and Safety Study (PLESS) showed that Qmax improved by 1.9 mL/s in the finasteride group in men with a mean prostate volume of 55cc, with a mean reduction in prostate volume of 18%.^24^ A similar effect has been confirmed with dutasteride, with a reduction in prostate volume of 23% after 6 months and 29% after 12 months.^25^ Similarly, the MTOPS and The Combination of Avodart and Tamsulosin (ComBAT) studies reported a 30% improvement in IPSS and Qmax with 5‐ARI monotherapy.^13^, ^26^

Dutasteride showed an improvement in Qmax of 2 mL/s, with an improvement in IPSS of five points.^27^ Both finasteride and dutasteride significantly reduce the risk of AUR (4.2%) and BPO‐related surgery (1%).^28^ The risk reduction is notable after 12 months of treatment and this becomes more pronounced with longer follow‐up to 4 years.^13^, ^26^

Analysis of population‐based administrative databases have reported that only 18% are still taking 5‐ARI at 1 year after commencing treatment, and this reduces to 16% at 2 years, 13% at 3 years, and 10% at 4 years.^11^

#### 2.2.2 Adverse effects

A meta‐analysis has shown that adverse effects of 5‐ARI are mainly related to sexual dysfunction (loss of libido and erectile dysfunction).^29^ A small proportion of patients will experience breast engorgement and gynecomastia due to the hormonal nature of the medication.^30^ 5‐ARI increase the serum estrogen levels and its usage has been potentially associated with an increased risk of breast cancer occurrence, although a systematic review did not find any evidence of increased breast cancer risk in males exposed to 5‐ARI.^31^, ^32^ Population‐based studies have suggested that long‐term use of 5‐ARI may be associated with an increased risk of cognitive decline, depression and self‐harm, and long‐term sexual dysfunction (even after cessation of treatment),^33^, ^34^, ^35^ and so patients should be counseled about these risks prior to commencing treatment.

#### 2.2.3 Special attention

##### Time to onset

5‐ARI reduce the disease progression in patients with large glands. Maximal clinical efficacy is experienced at 6 months.^13^ However, patients must continue the therapy indefinitely to experience the clinical benefits; after cessation of therapy the reduction in prostate volume and PSA start to recover to baseline levels from 3 to 6 months.^36^

##### Hematuria and non‐urological indications

An off‐label use of 5‐ARI is for the management of hematuria secondary to an enlarged prostate gland. Treatment with 5‐ARI has demonstrated decreased prostate expression of vascular endothelial growth factor (VEGF) thus effecting gland vasculature, with some evidence that it reduces bleeding during TURP.^37^, ^38^ 5‐ARIs are also used for reducing male pattern baldness and as a means to suppress the androgen levels after gender reassignment surgery, particularly in patients who desire a minimal or slower transition.

##### Prostate‐specific antigen (PSA)

5‐ARI therapy reduces the serum PSA values by up to 50%, but the levels may fluctuate widely. This should be considered when monitoring PSA in patients on 5‐ARIs and observing the trend of PSA change may be more beneficial in this group.^39^

### 2.3 Combination therapy

α1‐antagonists and 5‐ARIs have different mechanisms of action thus its combined use has synergistic effects. α1‐antagonists have a rapid onset of action, whereas 5‐ARIs have a longer duration to maximal efficacy.

#### 2.3.1 Efficacy

Long‐term data from the MTOPS and ComBAT trials showed significant reduction in IPSS when compared to monotherapy alone (6.3 points vs 3.8 points for tamsulosin alone and 5.3 points for dutasteride alone). Additionally, it also showed significant reduction in the risk of AUR or BPO‐related surgery compared to monotherapy alone.^13^, ^26^

Adherence to treatment is greater with combination therapy than for either agent alone, with reported adherence rates of 9% at 1 year after commencing treatment, 7% at 2 years, 5% at 3 years, and 4% at 4 years.^11^

#### 2.3.2 Special attention

After a 6‐month period of combination therapy, careful consideration is required before withdrawing α1‐antagonist therapy. Although symptom relief is maintained in the majority following α1‐antagonist withdrawal, patients with severe symptoms of BPO (IPSS ≥ 20) may benefit from a longer duration of combination therapy.^40^

### 2.4 Phosphodiesterase‐5 inhibitors (PDE‐5i)

The entire lower urinary tract expresses PDE‐5.^41^ PDE‐5i increases the intracellular cyclic guanosine monophosphate. There are many hypothesized mechanisms of how PDE‐5i improve the symptoms of BPO, including an increase in lower urinary tract oxygenation, smooth muscle relaxation, and regulation of lower urinary tract‐related inflammation.^42^ The exact mechanism of action of PDE‐5i in BPO remains unclear.

#### 2.4.1 Efficacy

Tadalafil is the only PDE‐5i approved for the treatment of BPO. Patients develop clinically significant improvement after 1 week and 4 weeks with tadalafil 5 mg once daily, with improvements in IPSS of 3 points and improvements in Qmax of 1.1 mL/s.^43^, ^44^ A significant reduction in bladder storage and voiding symptoms during treatment was also noted, although there was no significant change in PVR or Qmax.^45^ Several trials have also suggested that the combination of PDE‐5i and α1‐antagonists significantly improves LUTS and erectile dysfunction in patients with BPO.^46^

#### 2.4.2 Adverse effects

PDE‐5is are relatively safe. A meta‐analysis reported that 1.1%‐1.9% develop severe adverse effects after PDE‐5i therapy.^47^ Flushing (4.4%), dyspepsia (3.7%), and dizziness (1.7%) were the more common side effects.^48^

### 2.5 Adherence and contributing factors

Pharmacological therapy in BPO is a success story in urological practice. It has turned a mainly surgical problem into a chronic medical condition. However, as for most chronic conditions, long‐term adherence to pharmacotherapy is typically low. In BPO, adherence rates are related to severity of symptoms with the higher the degree of BPO the higher the adherence rate.^14^ A large population‐based cohort study of an administrative database of 1.5 million men aged over 40 years old treated with α‐antagonists, 5‐ARI, or combination therapy, demonstrated low persistence rates at 1 year of 35%, 18%, and 9%, for α‐antagonists, 5‐ARI, and combination therapy, respectively.^11^ Importantly, discontinuation was highest for the combination therapy and discontinuation was an independent risk factor for the hospitalization for BPO and BPO surgery (HR 1.65 & 2.80, *P* < .0001).^11^ Apart from lack of efficacy and adverse events, other common reasons affecting discontinuation of treatment include treatment regime change (19.8%), surgical intervention (6.2%), and improvement in BPO symptoms (8.5%).^49^ Furthermore, with long‐term pharmacotherapy for BPO there are increasing reports of associations with neurocognitive decline, depression, and long‐term sexual dysfunction.^2^ These factors all need to be considered when counseling men for pharmacological therapy. Although several newer pharmacological agents have been developed, these have not been shown to be any more effective or safe than older α‐antagonists or 5‐ARI.^50^ However, there are certain subgroups of patients who may benefit form a particular treatment (eg, 5‐ARI in those with larger prostate volume or PDE‐5i for men with both LUTS and ED) and this requires careful discussion on an individualized basis incorporating the patient's values and preferences.

## 3 MINIMALLY INVASIVE SURGICAL TREATMENTS (MIST)

### 3.1 Prostatic urethral lift (PUL)

**FIGURE 1:**
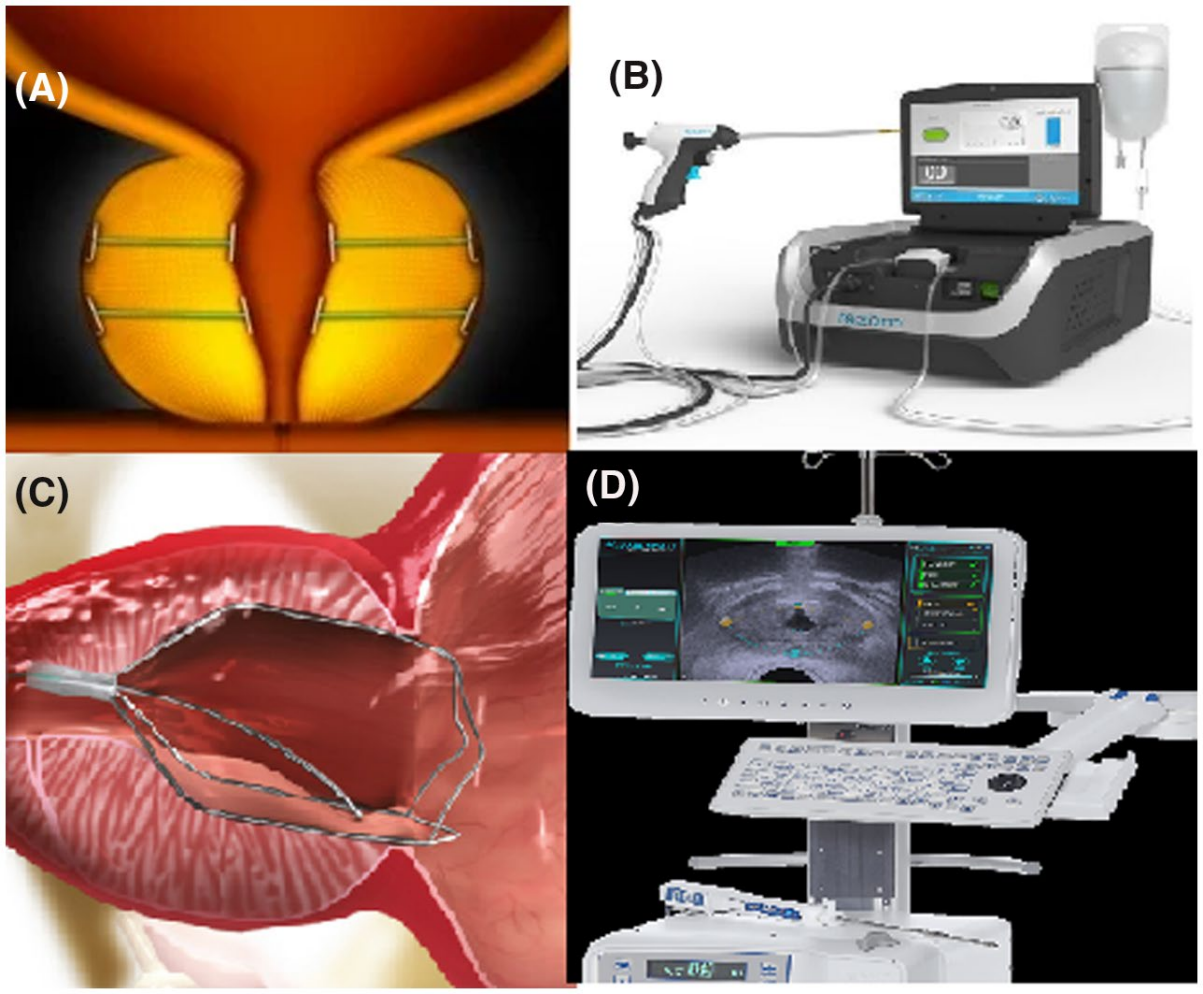
Minimally invasive surgical treatments. (a) Urolift; (b) Rezum; (c) TIND; (d) Aquablation

PUL (UroLift® System, NeoTract‐Teleflex, Pleasanton, CA, USA) is a relatively novel MIST in the management of patients with BPO (Figure 1a). The principle of the procedure involves utilizing permanent implants, which consists of two nitinol stainless steel anchors connected together by a non‐absorbable polyethylene terephthalate suture. One of the nitinol anchors is placed at the prostatic capsule, and the other on the urothelium of the prostatic urethra. The mode of action is to compress the obstructing lateral prostatic lobes, causing tissue‐retraction and subsequently mechanical urethral expansion without tissue ablation or injury. Preclinical studies on canine models also suggested that PUL induces acute ischemia leading to tissue remodeling and focal atrophy at compressed regions.^51^

The procedure can be carried out under a short general or regional anesthesia, although the procedure could also be carried out under local anesthesia in an ambulatory setting, usually together with a combination of sedatives and a prostatic block.^52^ Under cystoscopic guidance, the PUL implants are deployed using a specific PUL delivery device. Implants are typically placed at the 2 and 10 o'clock positions anterolaterally. This technique aims to compress the anterior third of the obstructive lobe, with implants placed from approximately 1‐1.5 cm distal to the bladder neck to the proximal aspect of the verumontanum.^51^ The number of implants required vary according to size and morphology of the prostate, commonly varied between 2 and 5 in total.^52^

#### 3.1.1 Efficacy

Two RCTs have been conducted to investigate the efficacy of PUL. The L.I.F.T. study was a multi‐center, double‐blinded, randomized trial, in which patients were allocated to PUL (n = 140) or sham procedure (n = 66) at a ratio of 2:1. At 3 month, patients in the PUL group had significant improvement in IPSS (*P* = .003), QoL (*P* < .001), Qmax (*P* = .005), and BPHII (*P* < .001) when compared to the sham‐controlled group.^53^ Improvement in terms of IPSS, QoL, BPHII, and Qmax was found to be durable at 5‐year follow‐up, with improvement of 36%, 50%, 52%, and 44%, respectively.^53^ The BPH6 study was a randomized‐controlled trial of PUL compared against TURP, which included 40 patients in each arm with 2‐year follow‐up.^54^, ^55^ Although significant LUTS relief was achieved in both arms of the study, TURP was found to be superior in terms of IPSS (−15.3 vs −9.2) and Qmax (+15.8 mL/s vs + 5 mL/s), but both BPHII and QoL were found to be similar between the two cohorts of patients.^55^

A meta‐analysis of five studies with a minimum follow‐up of 24 months showed favorable symptomatic improvement with PUL. IPSS improvement was found to be 9.1 in the randomized studies and 10.4 in the non‐randomized studies. Improvement in Qmax was 3.7 mL/s in the randomized cohort, and 3 mL/s in the non‐randomized cohort.^56^

#### 3.1.2 Periprocedural safety

The L.I.F.T. study found that 32% of patients developed postoperative urinary retention. For those who require a catheter following PUL procedure, the mean catheterization duration was 1 day.^53^ This is likely due to the fact that the implants are able to mechanically hold the prostatic urethra opened during the anticipated postoperative initial edema period. Blood transfusion postoperatively was rare, only one patient was reported to have developed a large pelvic hematoma 4 days after implantation with a significant drop in hemoglobin level.^57^ Otherwise side‐effect profile of PUL appeared to be mild, including transient dysuria, pelvic discomfort, mild self‐limiting hematuria, and urgency urinary incontinence, all resolve within 3‐weeks postoperatively. No postoperative stress urinary incontinence has been reported. However, the presence of PUL metallic implants may potentially have an impact on the quality of magnetic resonance imaging (MRI) at a later stage if the patient requires investigation for suspected prostate cancer, although there is a lack of study to investigate this at present.

#### 3.1.3 Functional outcomes

An attractive advantage of PUL is that it has been shown to be able to provide rapid improvement in symptoms, Qmax, and QoL, and patients were able to return to preoperative physical activity within 8.6 days.^53^ Another advantage of PUL is its ability to preserve both erectile and ejaculatory functions which appeared to be durable, with no de novo retrograde ejaculation identified in the meta‐analysis.^56^

#### 3.1.4 Durability and long‐term efficacy

In the BPH6 study, 11% of patients in the PUL arm required re‐intervention, compared with 6% in the TURP arm at 2 years. Surgical treatment was required in 13.6% in the L.I.F.T. study at 5 years. A recent systematic review and meta‐analysis of data from over 2000 patients reported an overall surgical re‐intervention rate of 6% per year, and this rate was higher for studies with longer follow‐up duration.^58^ Care must be taken during re‐intervention surgery especially during the morcellation phase if a laser enucleation technique is used, as the metallic implants may potentially damage the blades of the morcellator.^59^

#### 3.1.5 Special conditions

##### Median lobe

Current available evidence regarding the efficacy of PUL, including patients recruited for both RCTs, are based on patients with enlarged lateral prostate lobes only. The MedLift study investigated the use of PUL in 45 patients with enlarged median lobe with a follow‐up period of 12 months.^60^ Implants were deployed anterior to the 4 or 8 o'clock position to affix the obstructing aspect of the median lobe laterally. This results in the reduction of the “ball‐valve” effect caused by the median lobe. At 1‐year follow‐up, significant improvements were observed in IPSS, QoL, BPHII, and Qmax. None of the patients reported device‐related adverse event, and both ejaculatory and erectile functions were preserved.^60^

##### Prostate size

A study including 23 patients with prostate volume of >80 g demonstrated significant improvement in AUA symptom score (AUASS) when compared to 51 men with <80 g prostates.^61^ However, in this series, 22% of men with prostatic volume of >80 g were found to have a median lobe which required resection or ablation. Having initially approved only for patients with small to medium prostate size of <80 g since 2013, the U.S. Food and Drug Administration (FDA) has expanded indication for the use of PUL to patients with up to 100 g in size in 2020.

##### Urinary retention

One retrospective study investigated the outcomes of PUL in men with catheter‐dependent urinary retention, with a mean follow‐up of 7.1 months. Of the 30 patients included, 25 (83.3%) became catheter free within 3 days following treatment.^62^ A more recent retrospective study of 165 retention patients treated with PUL reported similar catheter‐free rates of 69% after 5 days, 83% by 1 month, and 87% at 2‐year follow‐up.^63^ Further surgical intervention was required in 8%. Early results from a prospective trial of PUL in 52 men with AUR and a mean prostate volume of 55cc was consistent with the previous studies, with a catheter‐free rate of 81% at 6 months.^64^

##### Anticoagulants

Currently, no studies are available in investigating the application of PUL in patients on antiplatelet anticoagulant therapy.

### 3.2 Convective radiofrequency water vapor thermal therapy (Rezum)

Convective radiofrequency water vapor thermal therapy using the Rezūm System (NxThera Inc.) is a novel minimally invasive surgical treatment (MIST) for benign prostatic enlargement (BPE) (Figure 1b). It is a water‐b

ased therapy system, which utilizes the convective Water Vapor Energy (WAVE) principle for the ablation of prostate tissue. The device has been approved by NICE in the United Kingdom.^65^

The Rezum system includes a power unit that generates thermal energy by condensation of the radiofrequency‐heated sterile water vapor. The generated energy is stored and delivered using a single‐use delivery system by deploying a fine curved transurethral needle. This needle punctures into the lateral or middle enlarged prostatic lobes under cystoscopic guidance to deliver the sterile vapor into the prostate. This large amount of energy is then dispersed rapidly through prostate tissue within the targeted prostatic zone (the transition zone) uniformly through the tissue interstices when injected at a slightly above interstitial pressure. This leads to disruption of cell membranes leading to cell death and tissue necrosis, closure of vasculature, and denervation of α‐adrenergic nerves.^66^ Subsequently, consolidation and reduction in prostatic size occurs over a few months.

Essentially the energy transferred is limited by the capsule of the targeted prostatic zones, and risk of injury to organs outside the prostate is extremely low. It is, therefore, able to target exactly the transition zone of the prostate, where the process of the development of BPH takes place utilizing a precise dose of stored thermal energy (540 cal/mL H_2_0). Each steam ablation cycle lasts for up to 9 seconds. Each treatment typically requires between 1 and 3 cycles to each prostate lobe, with a reported average of 4.6 applications required per procedure, and an average operating time of 8 minutes.^67^, ^68^

#### 3.2.1 Efficacy and durability

The effectiveness Rezum has been validated in a study using perioperative prostate MRI assessment. In this study, all prostates had at least 33% reduction in overall prostate and transition zone volumes at 3‐6 months following surgery.^69^, ^70^

Long‐term outcomes of a cohort of 188 men with moderate‐to‐severe LUTS treated with Rezum and followed up for 4 years revealed improvement of IPSS of 10.1 points (46.7%), Quality of Life (QoL) score of 2.0 points (42.9%), and Qmax of 4.2 mL/s (49.5%).^71^ The same study demonstrated a re‐treatment rate of 4.4% at 4 years. Other prospective clinical trials, perhaps able to provide real‐world perspective of Rezum as they include older patients and those with larger prostates, also supported these findings with improvement in IPSS of 50%‐60%, Qmax of 4‐6.3 mL/s.^67^, ^68^, ^72^

#### 3.2.2 Periprocedural safety

Rezum can be performed under general or spinal anesthesia, or as an office‐based procedure under local anesthesia. Patients are typically left with a urethral catheter postoperatively for 3‐7 days, and a trial without catheter is usually arranged a few days later depending on the size of the prostate treated. The use of a temporary prostatic stent for 2‐5 weeks following the procedure has also been reported to avoid the need for a postoperative urethral catheter.^68^ The long‐term study of Rezum reported the following complication rates: dysuria (16.9%), self‐resolving hematuria or hematospermia (11.8%), frequency and urgency (5.9%), AUR (3.7%), and suspected UTI (3.7%), which were either treated or resolved within 3 weeks.^71^ However, other studies have reported higher rates of UTI of up to 20% and urinary retention rates of up to 34%.^72^

Interestingly, a cost‐analysis study suggested that Rezum was comparatively effective in terms of health and financial burden when compared with TURP. This was largely due to fewer adverse events associated with Rezum. The study also demonstrated that Rezum therapy was cheaper to deliver when compared with other alternative MISTs such as Urolift.^73^

#### 3.2.3 Functional complications

No treatment‐related de novo erectile dysfunction was identified from the randomized‐controlled trial following Rezum and patients were able to preserve their ejaculatory functions with International Index of Erectile Function (IIEF) and Male Sexual Health Questionnaire (MSHQ) scores reported to remain unchanged for 2 years following treatment.^72^

#### 3.2.4 Special situations

##### Enlarged median lobe

Rezum has demonstrated efficacy in treating patients with enlarged median lobes. In the RCT, 30.9% of patients treated were known to have a median lobe, and efficacy was demonstrated to be equivalent to patients without a median lobe.^71^

##### Prostate size

At present, only patients with prostate size between 30 and 80cc are recommended for Rezum, although previous studies have demonstrated applicability of Rezum in treatment of patients with larger prostates of up to 120cc.^74^, ^75^ An on‐going multi‐center, single‐arm study (Rezum XL) will attempt to establish the efficacy in BPO patients with a prostatic volume between 80 and 150cc.^76^

##### Acute urinary retention

A recent study of 30 patients who were catheter‐dependent following urinary retention reported that 77% of the patients were able to void spontaneously following treatment with Rezum. The cohort of patients had a median age of 76 years, and the mean time to catheter independence was 29 days.^77^

##### Anticoagulants

The safety of Rezum in patients continuing antiplatelet or anticoagulant medication has not been investigated.

### 3.3 Temporary implantable Nitinol Device (TIND)

The temporary implantable nitinol device, TIND (Medi‐Tate™; Medi‐Tate Ltd., Or Akiva, Israel), is implanted cystoscopically and is designed to remodel the bladder neck and prostatic urethra through ischemic tissue necrosis and permanent mucosal incisions (Figure 1c). It is made entirely of nitinol, with the newer second‐generation device, the iTIND, comprising only three (as opposed to 4) elongated struts and anchoring leaflet.

#### 3.3.1 Efficacy

The first‐ and second‐generation devices have been studied in two prospective cohort studies in men with moderate LUTS and small prostates (mean prostate volume 29.5‐40 cm^3^).^78^, ^79^, ^80^, ^81^ Efficacy analysis revealed mean improvements in IPSS scores of 52%‐61% and Qmax of 57%‐101% at 12‐month follow‐up, but the lack of control arms in these studies limits the conclusions that can be reached.^79^, ^80^ There was no measurement of prostate volume post‐procedure, but it is likely that there is no debulking effect with this procedure.

#### 3.3.2 Periprocedural safety

The procedure is performed under sedation as a day case. Patients do not routinely require postoperative catheterization, and the patient returns for device removal under local anesthesia 5 days later. A low rate of significant periprocedural complications has been reported, with transient hematuria and dysuria commonest (7.4%‐12.3%), and 10% developing urinary retention.^79^

#### 3.3.3 Functional complications

There have been no reports of de novo ejaculatory or erectile dysfunction, but standardized tools were not used for assessment.^79^, ^80^ Similarly, there has been no evidence of incontinence or urethral stricture at 2‐year to 3‐year follow‐up.^78^, ^81^

#### 3.3.4 Durability

Evidence for durability is lacking. Two‐year follow‐up of the second‐generation device has shown maintained efficacy, with mean improvements in IPSS of 59% and Qmax 110% (compared to 60% and 101% at 1‐year follow‐up, respectively); however, a significant proportion (20%) was lost to follow‐up and 10% required further unspecified surgical intervention.^78^

#### 3.3.5 Special situations

##### Enlarged median lobe

Patients with enlarged median lobes were excluded from the studies of this device. Of those inadvertently recruited, the highest failure rate was in patients with enlarged median lobes (85.7% failure rate at 2 years), and so this should be considered a contra‐indication.^78^

##### Prostate size

This procedure has only been studied in those with small prostates (<65 cm^3^) and so there is no evidence for use in patients with larger prostates.

##### Acute urinary retention

These studies excluded patients with post‐void residual volume > 250 ml, or those in urinary retention, and so there is no evidence for this indication at present. Studies of iTIND for AUR are ongoing.^82^

##### Anticoagulants

Patients on anticoagulant or antiplatelet medication were excluded from the studies of this device and so there is no evidence that iTIND is safe in these patients.

### 3.4 Prostate artery embolization (PAE)

Prostate artery embolization (PAE) is a minimally invasive interventional radiological procedure utilizing embolic spheres to occlude the prostatic arteries leading to ischemic necrosis, predominantly of the central gland of the prostate.

#### 3.4.1 Efficacy

A recent systematic review and meta‐analysis of 13 studies (1254 patients) reported improvements in subjective and objective efficacy parameters at 6‐month follow‐up.^83^ The IPSS score at 12‐month follow‐up had improved by a mean of 69%, with a mean improvement in Qmax of 89% and post‐void residual of 77%. The mean reduction in prostate volume was 26%. In this meta‐analysis, the mean prostate volume at baseline was 77.3 cm^3^ (95% CI 71.4‐83.2). A more recent registry‐based study (UK‐ROPE study) confirmed this efficacy data in patients who had a larger mean baseline prostate volume of 101.2 cm^3^ (SD 57.2), with mean improvement in IPSS of 51%, Qmax of 50%, and mean reduction in prostate volume of 29 cm^3^.^84^

In terms of comparative data, a meta‐analysis of three randomized‐controlled trials and two large observational series (708 patients) of PAE against standard surgical treatments (TURP or open prostatectomy), reported greater efficacy with standard surgical treatment.^85^ The weighted mean differences between the groups were 3.80 for the IPSS (95% CI 2.77‐4.83), 3.62 ml/s for Qmax (95% CI 2.90‐4.34), 11.51 cm^3^ for prostate volume (95% CI 6.11‐16.91), and 1.02 ng/ml for PSA (95% CI 0.64‐1.40), all in favor of standard surgical treatment.

#### 3.4.2 Periprocedural safety

PAE is typically performed as a day‐case procedure under local anesthesia ± sedation, with a procedure time of 84‐144 minutes; periprocedural morbidity is low, with no requirement for catheterization, and time to return to normal activity is quicker with PAE compared to TURP (5 days vs 14 days).^84^, ^85^ A systematic review has identified major complications in only three patients (0.2%), due to UTI requiring intravenous antibiotics, bladder wall ischemia, and persistent perineal pain.^83^ In this review, rates of post‐procedural urinary retention and groin hematoma were 4.5% and 1.5%, respectively. A large cohort study reported Clavien‐Dindo grade I complications in 4.3% (groin hematoma, local arterial dissection, and penile ulceration) and Clavien‐Dindo grade II complications in 1% (one patient with sepsis and one groin hematoma requiring transfusion). Hematuria was significantly lower after PAE compared to TURP (18.6% vs 63.9%).^84^, ^86^

However, PAE can be associated with highly variable radiation exposure, despite technical modifications, with a randomized trial reporting a dose area product (DAP) of 176.5 Gy/cm^2^, and the UK‐ROPE registry reporting a DAP of 221 Gy/cm^2^.^84^, ^87^ The preoperative work‐up requires a CT‐angiography of the prostatic arteries which requires adequate renal function and also incurs a considerable dose of radiation exposure.

#### 3.4.3 Functional complications

In terms of sexual complications, PAE is associated with a lower risk of ejaculatory dysfunction with rates of 0.72% reported in a meta‐analysis. A rate of 24% was reported in the UK‐ROPE study (compared to 47.5% with TURP), but this is likely significantly overestimated as a large proportion of patients had experienced this prior to PAE secondary to medication use.^83^, ^84^ There is no overall significant change in sexual function after PAE, with stable IIEF scores at 1‐3 years’ follow‐up.^83^, ^84^ Furthermore, only two cases of urinary incontinence have been reported and the avoidance of transurethral instrumentation prevents risks of urethral stricture or bladder neck contracture.

#### 3.4.4 Durability

There is limited long‐term data for PAE. A large retrospective cohort study of 630 men revealed medium (1‐3 years) and long‐term (3‐6.5 years) cumulative success rates of 81.9% (95% CI 78.3%‐84.9%) and 76.3% (95% CI 68.6%‐82.4%), respectively.^88^ However, there was a significant loss to follow‐up and a re‐operation rate (repeat PAE, TURP, or open prostatectomy) of 11%. The UK‐ROPE registry reported that 20% of patients treated with PAE required further BOO surgery in the short term.^84^

#### 3.4.5 Special situations

##### Enlarged median lobe

The UK‐ROPE registry reported that 9 out of 43 patients that required re‐operation had median lobe enlargement, 4 had small prostate volumes, and 2 also had a high bladder neck, suggesting that PAE is more effective for those without enlarged median lobes.^84^ However, evidence for efficacy of PAE in patients with enlarged median lobes is mixed. In a small study of 18 patients with an intravesical prostatic protrusion (IPP) of > 5 mm, PAE led to reduction of mean IPP from 1.57 to 1.3 cm at 3‐month follow‐up with significant improvements in voiding LUTS.^89^

##### Prostate size

PAE has been shown to be more effective in larger glands. A post hoc analysis of a randomized trial suggested that a total prostate volume of 39 cm^3^ and adenoma volume of 38 cm^3^ was the optimal threshold to predict PAE success, and another study comparing medium sized (50‐80 cm^3^) to large prostates (>80 cm^3^) revealed greater efficacy in those with larger prostates with mean improvements in IPSS of 14 (SD 6.5) vs 10.5 (SD 5.5), Qmax of 6.0 mL/s (SD 1.5) vs 4.5 mL/s (SD 1.0), and prostate volume reduction of 42.3% vs 28.9%.^90^, ^91^

##### Acute urinary retention

Several studies have demonstrated efficacy of PAE in treating patients in urinary retention, with catheter‐free rates of 70%‐91% at short‐term follow‐up (typically within the first 3 months) in patients with mean prostate volumes of 70‐167 cm^3^.^92^, ^93^

##### Anticoagulants

Although PAE has not been extensively studied in patients on anticoagulants, one study of elderly comorbid patients, of whom 15% were taking clopidogrel, did not show any difference in the rates of bleeding complications, although this subgroup was not analyzed separately. PAE is likely to be safe in patients on antiplatelet or anticoagulant agents if radial artery access is possible, but if groin access must be used then closure devices (eg, Angio‐Seal) may need to be utilized.

## 4 SURGICAL TREATMENTS

### 4.1 Transurethral resection of the prostate (TURP)

Despite the development of several minimally invasive and novel technologies for the treatment of BPO in recent years, transurethral resection of the prostate (TURP), monopolar and bipolar, remains the standard to which all treatments are compared for prostate volumes <80 cm^3^.^94^

#### 4.1.1 Efficacy

Numerous systematic reviews have confirmed the efficacy of monopolar TURP. A meta‐analysis of 20 randomized trials with long‐term (5 year) follow‐up reported overall improvements in mean IPSS of 70%, Qmax of 162%, and PVR of 77%.^95^ Debulking is satisfactory with a mean of 54% reduction in prostate volume.^96^

A recent Cochrane review of 59 randomized trials with 8924 participants comparing bipolar to monopolar TURP has reported equivalence in terms of efficacy, with no significant difference in improvements in IPSS, Qmax, PVR, or extent of debulking at long‐term follow‐up.^97^

#### 4.1.2 Periprocedural safety

Large population‐based studies have revealed contemporary early morbidity and mortality rates of 11% and 0.1%, respectively.^98^, ^99^ Meta‐analysis has reported the following complication rates for TURP: bleeding requiring transfusion 2% (0%‐9%), TUR syndrome 0.8% (0%‐5%), AUR 4.5% (0%‐13.3%), clot retention 4.9% (0%‐39%), and UTI 4.1% (0%‐22%).^95^ Mean duration of catheterization is 2.5 days and mean hospitalization is 3.6 days.^96^ Bipolar TURP is safer, with lower rates of transfusion, clot urinary retention, and TUR syndrome.^95^

#### 4.1.3 Functional complications

A meta‐analysis of 30 studies reported the rates of ejaculatory dysfunction of 66%, with no significant difference between monopolar and bipolar technologies.^95^, ^100^ Rates of erectile dysfunction vary widely due to heterogeneity in measurement tools and confounding factors, but a review of 29 randomized trials has reported a mean rate of erectile dysfunction of 6.5%.^101^

Rates of long‐term bladder neck contracture (3%‐5%), urethral stricture (4%), and stress urinary incontinence (0.5%) were similar following monopolar and bipolar TURP.^95^, ^101^, ^102^ Reports of increased stricture or bladder neck contracture rates with specific bipolar devices require further confirmation in well‐designed long‐term studies.^103^, ^104^

#### 4.1.4 Durability

There is no other surgical procedure for BPO that has the demonstrable long‐term durability of TURP. Several cohort studies have reported durable and maintained efficacy in the majority at 8‐ to 22‐years’ follow‐up, with re‐operation rates of approximately 6%‐15% at 8‐to 10‐years’ follow‐up.^101^, ^105^, ^106^, ^107^ This was confirmed in large population‐based observational study of 41,059 men who underwent TURP, with a re‐intervention rate of 12.7% at 8‐year follow‐up, and the rate of repeat TURP has been reported at 1%‐2% per year.^1^, ^98^

Randomized trials with 5‐year follow‐up have confirmed equivalent long‐term efficacy between monopolar and bipolar TURP.^108^

#### 4.1.5 Special situations

##### Enlarged median lobe

TURP is effective in patients with enlarged median lobes. Furthermore, low‐level evidence suggests that “middle‐lobe only” TURP can provide durable efficacy with reduced rates of ejaculatory dysfunction and long‐term follow‐up.^109^

##### Prostate size

Randomized trials for men with larger prostates (>80 mL) are lacking, but large observational studies have reported that rates of bleeding requiring transfusion, postoperative sepsis, and mortality increase with larger prostate resection weights and longer operative times.^99^, ^110^ Therefore, TURP is only recommended for prostate volumes of 30‐80 cm^3^.^1^ However, bipolar enucleation of the prostate has been studied in larger prostates with comparable outcomes to Holmium laser enucleation of the prostate (HoLEP), and a systematic review has reported greater efficacy and safety compared to monopolar TURP.^111^, ^112^

##### Acute urinary retention

TURP is effective in treating AUR, but it has been reported that patients with long‐term indwelling catheters have higher rates of perioperative UTI, sepsis, and re‐catheterization.^113^

##### Anticoagulants

There is minimal evidence regarding the outcomes of TURP in anticoagulated patients, and all studies cease anticoagulation therapy peri‐operatively. The evidence is contradictory with some studies showing no increased risk of cardiovascular or cerebrovascular events, or bleeding‐associated morbidity, whereas others report higher rates of transfusion, secondary hemorrhage, and thromboembolic events.^114^, ^115^, ^116^ TURP should not, therefore, be performed in patients who must continue their anticoagulation.

### 4.2 Aquablation

Aquablation (Procept BioRobotics) is a new surgical treatment using the Aquabeam® device (Figure 1d). This technique combines the use of ultrasound image guidance and autonomous robotics, removing prostatic tissue using a heat‐free high‐velocity saline jet transurethrally. The patient requires either general or spinal anesthetics.

The Aquabeam® system consists of three main components—the central processing unit (CPU), a console, and a robotically guided 24F handpiece. During the procedure, with the patient placed at a dorsal lithotomy position, a custom‐made cystoscope is introduced transurethrally and view of the prostatic urethral lumen is obtained. The cystoscope, along with the handpiece, is positioned such that the tip is located at the bladder neck, and proximal to the external urethral sphincter. This is then secured with the unit's articulating robotic arm. A biplane transrectal ultrasound (TRUS) with both transverse and sagittal views are mounted in position to acquire live TRUS images of the prostate, which are displayed by the CPU. The operator is then required to determine the area of resection following contouring of the prostate. This includes the identification of crucial landmarks with regards to preservation of continence and ejaculatory functions.

Aquablation is then initiated, with a pump generating a high‐velocity sterile saline stream at a 90‐degree angle, with its rotational and longitudinal movement of the hand‐piece probe automatically controlled by the console according to the prescribed resection plan. The flow rate of the saline jet is based on the depth of the penetration required, ablating prostatic tissue. Hemostasis is secured using a urethral catheter on traction, although the use of diathermy hemostasis of the resection bed may also be required.^117^

#### 4.2.1 Efficacy and durability

Following the initial report on the feasibility of Aquablation in treating patients with BPE in 2016,^117^, ^118^ a RCT was conducted to investigate its efficacy and safety. The WATER study was a multi‐center, international, double‐blinded RCT comparing Aquablation with TURP across 17 sites at a ratio of 2:1.^119^ Patients with prostate volume of less than 80cc were included. One hundred and eighty‐one men were randomized to the Aquablation group and 67 to TURP group. At 2 years, the improvement in IPSS (14.7 vs 14.9, *P* = .83) and Qmax (11.2 vs 8.6, *P* = .188) were similar between both groups. The mean resection time was significantly less with Aquablation when compared to TURP (4 vs 27minutes), although a variable set up time is usually required when setting up and contouring prior to resection when using Aquablation. The automated ablation principle used means that the resection time and intraoperative irrigation fluid requirement is significantly less than that of TURP. Other prospective single‐armed studies also had similar findings.^118^, ^120^ However, Aquablation is still at its infant stage, and long‐term efficacy data are still lacking.

#### 4.2.2 Periprocedural safety

Regarding the primary safety endpoint, which was defined as the occurrence of persistent Clavien‐Dindo grade 1 or grade ≥2 surgical complications, Aquablation showed a significantly lower rate of occurrence compared to TURP (26% vs 42%, *P* = .015) in the WATER study.^119^

In early studies, one of the major concerns with Aquablation was hemostasis. For smaller prostates this did not appear to be significant with a mean hemoglobin drop of 5.7% postoperatively.^118^ In order to achieve hemostasis, various techniques were employed, including deploying a balloon catheter inflated in the prostatic fossa following ablation or placing specially designed double‐balloon catheter‐tensioning device (CTD) developed by PROCEPT BioRobotics under traction overnight postoperatively.^121^ However, despite this, hemostasis appeared to be more significant when ablating larger prostates. In the WATER II study, which included patients with a mean prostate volume of 107cc, mean hemoglobin drop was 2.9.^122^ Ten of the 101 patients (10%) required a postoperative blood transfusion while 5 patients needed to return to operating theatre for hemostasis by fulgurations. However, with increasing experience hemostasis has improved and the CTD device is no longer routinely used. A more recent multi‐center study of 801 patients reported overall transfusion rates of 1.4%‐2.5% when standard traction and selective bladder neck cautery was used, although rates were higher when larger prostates were treated.^123^

Aquablation requires either a general or spinal anesthesia and is not feasible to be done as an office‐based procedure at present.

#### 4.2.3 Functional complications

Antegrade ejaculation, even when treating large prostates, was preserved in 81% of sexually active patients in the WATER II study, with no change in IIEF‐15 domains compared to baseline.^124^

#### 4.2.4 Special situations

##### Median lobe

The presence of a median lobe is not a contraindication when using Aquablation to treat BPO, as long as it is contoured during the pre‐planning phase of the procedure.^117^, ^120^

##### Prostate size

The WATER II trial was conducted to prospectively assess the feasibility and safety of Aquablation in treating 101 patients with larger prostates between 80cc and 150cc (mean 107cc).^125^ Efficacy was similar to that reported for smaller prostates and operating time was relatively short despite the large prostate sizes included in the study, with a mean time of 37 minutes only and a resection time of 8 minutes, although some patients required additional Aquablation passes to complete ablations. However, as previously discussed, hemostasis is a concern and a 2% rate of de novo incontinence was reported at 1‐year follow‐up.

##### Anticoagulants

As hemostasis could be challenging, especially for patients with larger prostates, patients who are on anticoagulants are required to be stopped prior to the procedure.

### 4.3 Photoselective vaporization of the prostate (PVP)

Photoselective vaporization of the prostate (PVP) utilizes the potassium‐titanyl‐phosphate (KTP) or lithium triborate (LBO) lasers (also known as “Greenlight lasers”) which work at a wavelength of 532 nm and are selectively absorbed by oxyhemoglobin, promoting coagulation and cell disintegration. Three “Greenlight” lasers have been studied, each with different maximum power outputs and fiber designs—the 80‐W KTP, 120‐W LBO, and 180‐W KTP.

#### 4.3.1 Efficacy

Randomized trials and large cohort series of the 80‐W KTP laser have revealed improvements in mean IPSS of 61%‐70%, Qmax of 121%‐172%, and PVR of 83%‐84% at 12‐month follow‐up, with changes in mean prostate volume of 23%‐41% and PSA of 44% in the short term.^126^, ^127^, ^128^ Similar data have been reported for the higher‐powered lasers, with improvements in mean IPSS of 73%, Qmax of 114%, and PVR of 100% for the 120‐W laser, and improvements in mean IPSS of 67%, Qmax of 141%, PVR of 61%, and mean reduction in prostate volume of 55% with the 180‐W laser.^129^, ^130^

In terms of comparative efficacy data, several meta‐analyses have shown equivalence between PVP and TURP (monopolar and bipolar) in terms of improvements in IPSS, Qmax, PVR, and prostate volume reduction.^131^, ^132^, ^133^

#### 4.3.2 Periprocedural safety

Pooled analyses of randomized trials and cohort studies have demonstrated the superior perioperative safety of the “Greenlight” lasers compared to monopolar TURP. Although operative time is longer (mean 9.37 minutes), there is a significantly lower rate of blood loss (mean difference 1.33 g/dL of hemoglobin), transfusion (0.2% vs 7.7%), clot retention (86% lower risk with PVP), TUR syndrome (81% lower risk with PVP), catheterization time (mean difference 32.4 hours), and hospitalization (mean difference 1.8 days) with PVP^95^, ^132^ compared to monopolar TURP. However, PVP is associated with a higher prevalence of short‐term dysuria (6%‐30%), more pronounced in those with smaller prostates, compared to monopolar TURP.^128^

#### 4.3.3 Functional complications

Rates of ejaculatory dysfunction after PVP range from 22% to 67% in the limited series that specifically report this outcome.^129^, ^130^, ^134^, ^135^ Meta‐analyses assessing sexual dysfunction based on IIEF scores have suggested no significant difference between PVP and TURP in terms of ejaculatory or erectile dysfunction, but the evidence is limited by the heterogeneity of assessment and measurement tools used between studies.^132^

Meta‐analysis and cohort studies have reported that rates of bladder neck contracture (4%), urethral stricture (4%), and stress urinary incontinence (1%‐3%) are equivalent to TURP in the short term (2‐year follow‐up).^129^, ^132^

#### 4.3.4 Durability

Long‐term follow‐up from randomized trials of PVP are lacking. Improvements in IPSS and Qmax are maintained at 3‐year follow‐up.^136^ A randomized trial of the 120‐W laser reported significantly higher re‐treatment rates with PVP (11% vs 1.8%) at 3‐year follow‐up; a randomized trial of the 180‐W laser reported a re‐treatment rate of 9% (compared to 7.6% with TURP) at 2‐year follow‐up, although the re‐intervention rate for TURP in this study was higher than expected.^129^, ^136^ A cohort study with up to 5‐year follow‐up reported an overall re‐intervention rate (modality of retreatment unspecified) with 80‐W PVP of 15%, and meta‐analysis has confirmed higher re‐treatment rates with PVP compared to TURP (RR 1.81).^128^, ^132^

#### 4.3.5 Special situations

##### Enlarged median lobe

PVP is an effective option in men with enlarged median lobes.

##### Prostate size

There have been no randomized trials of PVP in men with large prostates, but several cohort studies have reported satisfactory outcomes in men with prostate volumes >80 cm^3^. A cohort study of 131 men revealed no difference in efficacy between prostate volumes of <40, 40‐80, or >80 cm^3^, and this has been confirmed in other series.^137^, ^138^ In another cohort of 54 men with prostate volumes >100 cm^3^ (mean 135 cm^3^), improvement in mean Qmax was sustained at 141% at 2‐year follow‐up, and efficacy has also been reported to be comparable in men with very large prostates (>200 cm^3^).^139^, ^140^ However, operative time and energy delivery are increased with larger prostates, and higher rates of conversion to TURP have been reported with the lower energy lasers, but there is no difference in catheterization time, hospitalization time, or longer‐term complications.^141^ Evidence of a higher re‐treatment rate in those with larger prostates is mixed, with one study reporting re‐treatment rates of 5.4% at 24 months and 9.3% at 36 months.^142^ This requires further evaluation in long‐term studies.

##### Acute urinary retention

PVP has been demonstrated to be effective in men with AUR, with reported catheter‐free rates of up to 96%, and no difference in long‐term complications to those not in urinary retention.^138^, ^143^

##### Anticoagulants

The reported advantage of PVP is its low risk of bleeding complications and demonstrated safety in patients on anticoagulant or antiplatelet agents, or who are deemed high anesthetic risk. A study of 33 men on aspirin, clopidogrel, or warfarin did not show an increased risk of bleeding or need for transfusion, and similar findings were seen in a larger study of 162 men which reported a delayed bleeding risk of only 4%.^138^, ^144^ Despite the lack of randomized trials, PVP appears to be safe for treating patients on anticoagulant or antiplatelet agents.^1^

##### Tissue for histopathological analysis

An important consideration in performing PVP is the lack of tissue specimen for histopathological analysis, as opposed to TURP or enucleation procedures. Therefore, patients should undergo thorough preoperative assessment with PSA (with MRI if the PSA is elevated) in order to exclude significant prostate cancer prior to treatment with PVP.

### 4.4 Anatomical Endoscopic Enucleation of the Prostate (AEEP)

First described by Haroka in 1983,^145^ the concept of anatomical endoscopic enucleation of prostate (AEEP) was later appreciated following the report by Gilling et al on their initial experience with HoLEP.^146^ HoLEP has since been the most popular and studied AEEP technique. The procedure is carried out typically using a high‐power holmium laser (100 W or 120 W), utilizing a reusable end‐firing 550‐micron laser fiber, although the use of lower‐power HoLEP has also been described with non‐inferior efficiency when compared with high‐power HoLEP.^147^ A 26F continuous‐flow endoscope using 0.9% saline irrigation fluid is required for the procedure, and the laser fiber is delivered through a laser bridge.

Although the “2‐lobe” and “en‐bloc” techniques have been described and have been gaining popularity in recent years,^148^, ^149^ the classical three‐lobe HoLEP technique involves incising the prostatic mucosa from 5 and 7 o'clock positions from the bladder neck toward the level of verumontanum. The two incisions are then joined together distally, with the median lobe subsequently dissected off the capsule retrogradely. This is achieved using the beak of the endoscope to mechanically retract the prostatic adenoma off the capsule mimicking the principle used in open Millin's prostatectomy. The laser is used to develop the plane and for hemostasis as the adenoma is being enucleated, maintaining a clear vision throughout the procedure.

The enucleated median lobe is then pushed into the bladder before being dislocated completely. The same technical principle is then applied for both the lateral lobes. Usually a small bridge of urethral mucosa is preserved anteriorly to reduce the risk of external sphincter injury, with some surgeons preferring to release this early on in the procedure to minimize traction‐related damage to the sphincter.^150^

The HoLEP endoscope is then exchanged with a nephroscope and a morcellator is then inserted via its straight working channel. The morcellator consists of blades which either oscillate or reciprocate to morcellate the free‐floating enucleated lobes in the distended bladder. The morcellated tissue can be sent for histopathology analysis, and a urethral catheter is then inserted prior to the end of the surgery.

Other energy sources have also been utilized in carrying out AEEP, including transurethral bipolar/plasmakinetic enucleation of the prostate (BipoLEP),^151^ transurethral Tm:YAG vapoenucleation,^152^ transurethral anatomical enucleation of the prostate with Tm:YAG laser (ThuLEP) which utilizes mechanical enucleation with laser for mucosa dissections,^153^ diode laser enucleation of the prostate (DiLEP),^154^ and Lithium Borate “Greenlight” enucleation of the prostate (GreenLEP).^155^ They all follow the same principles of AEEP, and it has been suggested that surgeon's preference, resource availability, and technical competency of the surgeon are perhaps more important than the energy source when performing AEEP.^156^

#### 4.4.1 Efficacy and durability

HoLEP has been highly scrutinized with level 1 evidence to suggest that it provides at least equivalent if not better improvement in terms of long‐term surgical outcomes when compared to TURP and other vaporization or laser techniques.^134^, ^157^, ^158^, ^159^, ^160^, ^161^ Cornu et al conducted a meta‐analysis of 69 RCTs including various modalities of BPO surgeries such as TURP, PVP, bipolar TURP, and HoLEP.^95^ In this study, HoLEP was demonstrated to be the only technique which offers a significantly greater improvement in IPSS, Qmax, and PVR when compared to the conventional monopolar TURP. A network meta‐analysis comparing the efficacy of TURP, PVP, Thulium laser enucleation of the prostate (ThuLEP), Thulium laser resection of the prostate (TmLRP) diode laser enucleation of the prostate (DiLEP), and diode laser vaporization of the prostate (DiLVP) also demonstrated that HoLEP ranked top in terms of IPSS and Qmax at 12 months. A recent RCT also failed to demonstrate any difference in efficacy or safety between TURP and Thulium laser vaporesection of the prostate (ThuVARP).^162^ The retreatment rate of HoLEP has been reported at 0% in a long‐term outcome study of an RCT with 7 years of follow‐up,^163^ compared with 1%‐2% per year when treated with monopolar TURP.^1^

A striking advantage of HoLEP is that it is highly effective regardless of prostatic volume, since HoLEP removes the entire transition zone adenoma of the prostate regardless of prostate size. Thus, its durability is independent of prostate size being treated. Kuntz et al divided 384 men into three groups (<40cc, 40‐79cc, and >79cc).^164^ No significant differences in American Urological Association (AUA) scores, Qmax, or post‐void residual (PVR) were detected on follow‐up between the three groups. This was supported by a similar study which included even larger prostates (>200cc), although catheter times appeared to be longer but transfusion rate, re‐catheterization, and complication rates remained the same.^165^

#### 4.4.2 Periprocedural safety

RCTs and meta‐analysis studies consistently reported less bleeding when compared with TURP.^95^ A meta‐analysis conducted by Wroclawski et al also demonstrated that HoLEP was associated with lesser reduction in hemoglobin level and blood transfusion rates when compared to other prostate resection techniques.^166^ This also appears to be the case regardless of size of prostate being treated.^164^ Less bleeding also subsequently leads to shorter postoperative catheter duration and hospital stays when compared with TURP.^166^

HoLEP is often perceived to be a difficult procedure to learn, in particular the release of anteroapical mucosal attachment of lateral lobes and apical enucleation. Morcellation has also been considered challenging with reported incidence of bladder mucosal injury of up to 18% in early series.^159^ It has been suggested that between 20 and 30 procedures were required to achieve consistent and reproducible results.^157^ However, this could be overcome with a structured mentorship program, with over 90% of mentees able to continue with HoLEP independently afterwards.^167^

#### 4.4.3 Functional complications

Postoperative stress urinary incontinence (SUI) has been a concern following HoLEP, but with the use of early apical release and preservation of distal anterior urethral mucosa, SUI is uncommon and is mostly transient after treating larger volume prostates.^150^ HoLEP has also been demonstrated to have no impact on overall erectile function.^168^

#### 4.4.4 Special situations

##### Median lobe

No safety issues have been reported to be associated with patients with large median lobe during HoLEP.

##### Acute urinary retention

When treating men with refractory urinary retention, HoLEP has been demonstrated in multiple studies to offer excellent catheter‐free rates following treatment. Of 169 patients with non‐neurogenic refractory urinary retention treated with HoLEP in a study conducted by Elzayat et al, 98.3% of the patients remained catheter‐free at 3‐year follow‐up.^169^ This was supported by two other retrospective studies with a catheter‐free rate of 99%‐100%, despite many patients having evidence of reduced detrusor contractility in addition to bladder outlet obstruction.^170^

##### Anticoagulants

Evidence from RCTs demonstrated that HoLEP has fewer bleeding‐related complications when compared with TURP, suggesting that HoLEP in patients on oral anticoagulants is comparatively safer.^157^, ^158^, ^159^, ^160^, ^170^ A retrospective comparative study of 76 patients included patients on coumadin and aspirin at time of surgery, with no statistical differences in bleeding complication rates between the groups.^171^ The study suggested that HoLEP is a safe alternative to TURP when treating patients on oral anticoagulants owing to its excellent hemostatic properties.

## 5 DISCUSSION

There are several pharmacological, interventional, and surgical treatment options for LUTS secondary to BPO, each with unique risks, benefits, and effects on patients’ quality of life. When counseling men regarding these options, urologists must incorporate individual patient's values and preferences to optimize decision making and enable personalized care.^172^ The principal factors that should be considered are shown in Table 4 and use of a personalized decision aid may facilitate this discussion.^173^

**TABLE 4:** Key factors to consider when counseling men regarding treatment for BPO

Patient factors	Prostate factors	Values and preferences
Age	Size	Sexual side effects
Comorbidity	Enlarged median lobe	Incontinence
Anticoagulant use	Treatment for voiding LUTS or for urinary retention	Postoperative length of catheterization
		Length of hospital stay
		Perioperative transfusion
		Need for re‐treatment
		Type of anesthetic
		Postoperative recovery period
		Experience of the surgeon

The initial consideration should be regarding a patient's preference and suitability for pharmacological vs interventional/surgical therapy. In elderly, comorbid patients who are deemed unsafe for general or regional anesthesia, pharmacological therapy or a MIST should be discussed. Risks of pharmacological treatment, especially with polypharmacy in the elderly population, should be considered, as well as the relative efficacy depending on the patient's most bothersome complaints (eg, LUTS vs urinary retention). For those who desire greater efficacy and are willing to have a procedure under local anesthesia, Rezum, Urolift, or PAE will provide greater symptom improvement to pharmacotherapy with greater durability. For men who desire a procedure with the greatest proven long‐term symptom improvement and who are not concerned about sexual function, TURP, PVP, and AEEP should be considered depending on prostate volume and bleeding risk (eg, those on anticoagulation are likely to have a lower bleeding risk with PVP that TURP). In those whose primary concern is regarding impact on sexual function, Urolift offers the lowest rate of sexual dysfunction (0%) and Rezum has very low rates of sexual dysfunction (0%‐3%), while pharmacotherapy has higher rates of sexual dysfunction than all MIST options. In those who desire a minimally invasive procedure under local anesthesia, Urolift, Rezum, and PAE should be considered, but patients should be informed about the radiation dose involved with the PAE planning and procedure, and the unknown long‐term risk of this.^174^ In those who desire a procedure without catheterization, Urolift and PAE remain good options. Prostate anatomy will influence the options that are deemed suitable, with PVP, Aquablation, and AEEP more suitable for larger prostates (>80cc). Open and robotic simple prostatectomy are also options for very large BPH, or in those who also have large bladder stones, but these options are not discussed in this review as they are not widely performed. In those with large median lobes who desire a MIST, Rezum is likely to provide greater symptom improvement to Urolift or PAE, but ongoing studies will help to clarify the efficacy of each option in this scenario.

There is no “one‐size‐fits‐all” treatment for LUTS due to BPO and management should be personalized based upon the patient's medical history, prostate anatomy, and individual values and preferences. Careful and thorough discussion regarding the key points described above is required to ensure optimal patient counseling and appropriate decision making, with the aim of improving patient satisfaction and reducing regret after treatment.

## 6 CONCLUSION

Over the last 10 years, several new treatments have been introduced to the market to treat voiding LUTS due to BPE causing BOO. These mainly consist of minimally invasive treatment trying to reduce the hospital stay and postoperative complications. To date, no treatment has withstood the test of time compared to TURP in terms of long‐term efficacy, except HoLEP; nonetheless, it is important to counsel patients appropriately regarding all available treatment options as patients may favor reduced efficacy in association with reduced side effects.

## CONFLICT OF INTEREST

The authors have no conflict of interest to declare related to this submission.
